# Efficacy and Response Biomarkers of Apatinib in the Treatment of Malignancies in China: A Review

**DOI:** 10.3389/fonc.2021.749083

**Published:** 2021-10-05

**Authors:** Zhichao Tian, Xiaohui Niu, Weitao Yao

**Affiliations:** ^1^ Department of Orthopedics, The Affiliated Cancer Hospital of Zhengzhou University and Henan Cancer Hospital, Zhengzhou, China; ^2^ Department of Orthopedic Oncology, Beijing Jishuitan Hospital, Beijing, China

**Keywords:** apatinib, tyrosine kinase inhibitor, efficacy, response biomarker, clinical trial

## Abstract

Apatinib is a multitarget tyrosine kinase inhibitor marketed in China for the treatment of advanced gastric cancer (GC) and hepatocellular carcinoma (HCC). It has also been used off-label for the treatment of many other malignancies. To comprehensively evaluate the efficacy of apatinib as a targeted therapy in the treatment of malignancies, we conducted systematic online and manual searches of the literature on apatinib in the treatment of malignancies. In this review, we first summarized the efficacy of apatinib against various malignancies based on clinical trials where results have been reported. In prospectively registered trials, apatinib has been proven to be effective against GC, HCC, lung cancer, breast cancer, sarcoma, esophageal cancer, colorectal cancer, ovarian cancer, cervical cancer, cholangiocarcinoma, diffuse large B-cell lymphoma, nasopharyngeal carcinoma, and differentiated thyroid cancer. The response biomarkers for apatinib were also reviewed. This review will serve as a good reference for the application of apatinib in clinical studies and the design of clinical trials.

## Introduction

Apatinib is a multitarget tyrosine kinase inhibitor (TKI) developed by Hengrui Pharmaceutical Company, China. The targets that apatinib can inhibit include VEGFR1 (IC_50_ = 70 nM), VEGFR2 (1 nM), c-RET (13 nM), c-KIT (429 nM), and c-SRC (530 nM). Current evidence suggests that apatinib has a limited ability to inhibit PDGFRα (IC_50_ > 1000 nM), EGFR (> 10000 nM), HER-2 (> 10000 nM), and FGFR1 (> 10000 nM) (1).

Apatinib was approved for use in China in 2014 for the treatment of advanced gastric adenocarcinoma or gastroesophageal junction adenocarcinoma that had progressed or relapsed after at least two previous systemic chemotherapy treatments (2). Since then, apatinib has been widely used in China not only for advanced gastric cancer (GC) but also for a wide range of other malignancies in an off-label manner (3). In December 2020, apatinib was approved for increased use in China for the treatment of patients with advanced hepatocellular carcinoma (HCC) who had previously failed or were intolerant to at least one first-line systemic therapy. To date, apatinib has shown promising activity against many malignancies (3, 4). However, no review articles extensively evaluating the efficacy of apatinib as a targeted therapeutic agent for the treatment of malignancies have been published in the past 3 years. Thus, in this review, we first performed systematic online and manual searches of the literature. Next, we summarized the results of all registered clinical trials of apatinib for malignancy treatment and then searched for studies on efficacy markers of apatinib. Finally, based on the review, we have discussed the current problems to be solved in the clinical application of apatinib and the future directions of clinical research.

## Efficacy of Apatinib in Different Malignancies

To fully evaluate the efficacy of apatinib as a targeted therapeutic agent in the treatment of malignancies, systematic online searching and hand searching were conducted. The period included for this search was from January 2005 to July 2021. The inclusion criteria were as follows: 1) trials with a registration number; 2) prospective phase I, II, or III clinical trials; 3) trials of apatinib in the treatment of malignancies; and 4) trials with complete data published in open-access magazines. The retrieved literature and clinical trials were classified according to histological type of disease.

### GC

GC was the first cancer to be tested when apatinib entered clinical trials (**Table 1**). Results from one of the earliest phase II trials of apatinib for metastatic GC showed that among 144 patients with metastatic GC who had experienced treatment failure with two or more chemotherapy regimens, those treated with apatinib showed improved progression-free survival (PFS) and overall survival (OS) (5). A subsequent phase III clinical trial showed that for the 267 enrolled patients with advanced gastric or gastroesophageal junction adenocarcinoma, for whom two or more prior lines of chemotherapy had failed, apatinib significantly prolonged median OS (6.5 months for the apatinib group *vs* 4.7 months for the placebo group) and median PFS (2.6 months for the apatinib group *vs* 1.8 months for the placebo group) compared with placebo (6). As a result, apatinib was approved for marketing in China in 2014 (2, 15). However, the two clinical trials have attracted criticism and skepticism. These criticisms include: 1) compared with the placebo group, the apatinib group showed limited improvement in survival of GC patients; 2) the analysis of the basic measures was insufficient, and in some of the parameters, there were large differences between the trial and placebo groups; 3) the toxicity analysis was insufficient, considering apatinib was highly toxic at a dose of 850 mg/day (16–19). However, these doubts have not affected the large-scale clinical use of apatinib in patients with advanced GC in China.

**Table 1:** Clinical trials on apatinib treatment for GC included in this review.

Type of disease	Year of publication	Drugs or therapies	Total number of patients	Clinical outcome	References
Chemotherapy-refractory advanced metastatic GC	2013	Placebo vs apatinib 850 mg/day vs apatinib 425 mg twice daily	144	The median PFS times were 1.40, 3.67, and 3.20 months, respectively. The median OS times were 2.50, 4.83, and 4.27 months, respectively	(5)
Chemotherapy-refractory advanced or metastatic ASGJ	2016	Apatinib 850 mg/day or placebo once daily	267	Median PFS (2.6 vs 1.8 months) and median OS (6.5 vs 4.7 months) were significantly improved in the apatinib group compared with those in the placebo group	(6)
Advanced GC	2020	Apatinib monotherapy or apatinib plus chemotherapy	737	Patients who received combination therapy achieved significantly longer median PFS (6.18 vs 3.52 months) and median OS (8.72 vs 5.92 months) compared with those who received monotherapy	(7)
Advanced or metastatic ASGJ	2020	Apatinib 500 mg/day	321	The ORR was 10.60%. The median PFS and OS were 4.0 and 8.2 months, respectively	(8)
Previously treated metastatic GC	2020	Apatinib 250 mg vs apatinib 425–500 mg vs apatinib 675–850 mg once daily	120	The median PFS and OS were 4.03 and 6.27 months, 4.33 and 7.43 months, and 2.87 and 7.87 months for the low-, mid-, and high-dose groups, respectively, and were comparable between the three dose groups	(9)
Taxane-resistant advanced GC	2020	Apatinib 850 mg/day in combination with weekly paclitaxel or the POF regimen	20	The ORR was 11.1%, the median PFS was 3.5 months, and the median OS was 4.7 months	(10)
Locally advanced GA	2020	Three preoperative cycles of S-1 (80–120 mg/day on days 1–14) and oxaliplatin (130 mg/m^2^ on day 1) and two cycles of apatinib (500 mg/day for 21 days) at 3-week intervals, followed by surgery	29	The ORR was 79.3%	(11)
Advanced metastatic GC	2020	Apatinib 500 mg/day, days 1–21; S-1 40 mg/m^2^ twice daily, days 1–14	30	The median PFS was 4.21 months, and the median OS was 7.49 months	(12)
Advanced metastatic alpha-fetoprotein-producing GC	2021	Apatinib monotherapy or apatinib plus other drugs	20	The ORR of apatinib was 10%, the median PFS was 3.5 months, and the median OS was 4.5 months	(13)
Advanced ASGJ	2021	Camrelizumab combined with chemotherapy followed by camrelizumab plus apatinib	48	The ORR was 58.3%, the median PFS was 6.8 months, and the median OS was 14.9 months	(14)

GC, gastric cancer; PFS, progression-free survival; OS, overall survival; ASGJ, adenocarcinoma of the stomach or gastroesophageal junction; POF, paclitaxel, oxaliplatin, and 5-fluorouracil; ORR, objective response rate; GA, gastric adenocarcinoma.

Subsequent clinical trial results revealed that apatinib showed acceptable efficacy and safety in real-world Chinese patients with metastatic GC (7, 8); low doses of apatinib (250 mg or 500 mg/day) were tolerated by and beneficial to patients with advanced GC who had been heavily treated previously (9), and a lower dose of apatinib achieved comparable OS and PFS with a higher daily dose of apatinib while maintaining better safety (20). Apatinib has been further tested in other therapeutic scenarios. The findings suggested that apatinib combined with paclitaxel-based chemotherapy might be effective and tolerable in patients with chemotherapy-refractory GC (10); apatinib combined with S-1 was not superior to other chemotherapy regimens as first-line therapy for advanced GC (12); apatinib plus neoadjuvant chemotherapy followed by resection in patients with locally advanced gastric adenocarcinoma showed favorable activity and manageable safety (11); apatinib showed promising efficacy and an acceptable safety profile in patients with advanced alpha-fetoprotein-producing GC (13). According to the results of a recent clinical trial, apatinib combined with chemotherapy and PD-1 inhibitor exerted encouraging antitumor activity and manageable toxicity as first-line therapy for patients with advanced or metastatic gastric or gastroesophageal junction adenocarcinoma (14). In general, apatinib is currently considered a third-line or higher-line treatment option for advanced GC in China and is being continuously tested in combination therapies and various treatment scenarios for GC.

### HCC

The clinical trials of apatinib for HCC have only been conducted in recent years (**Table 2**). Results from a clinical trial in 2017 showed that transcatheter arterial chemoembolization (TACE) in combination with apatinib was superior to TACE alone in the long-term treatment of advanced HCC. The combination of TACE with apatinib can prolong the PFS of patients and has confirmed safety (21). In 2020, a phase III clinical trial of apatinib for advanced HCC showed that apatinib significantly improved OS in patients with pretreated advanced HCC compared to placebo (median 8.7 *vs*. 6.8 months, respectively), with a manageable safety profile (26). This result led to the approval of apatinib by the China Food and Drug Administration in late 2020 as an agent for additional indications in advanced HCC. Other clinical trials also confirmed the efficacy and safety of apatinib in advanced HCC (22, 23). Apatinib combined with a PD-1 inhibitor showed promising efficacy and manageable safety in patients with advanced HCC both in the first- and second-line settings, and apatinib at a dose of 250 mg was recommended as a combination therapy for advanced HCC treatment in further studies (24, 25). Based on the above clinical trial results, apatinib combined with PD-1 inhibitors has the potential to replace apatinib monotherapy in the treatment of advanced HCC (27). With its approval for the treatment of advanced HCC, apatinib will be further studied in the treatment of HCC. At present, the importance of apatinib in the treatment of HCC is significantly superior to its importance in GC treatment.

**Table 2:** Clinical trials on apatinib for HCC treatment included in this review.

Type of disease	Year of publication	Drugs or therapies	Number of patients	Clinical outcome	References
Intermediate and advanced HCC	2017	TACE alone *vs* TACE with apatinib	44	The median PFS was 6.0 months in the TACE group and 12.5 months in the TACE with apatinib group, with significant difference	(21)
Advanced HCC	2020	Apatinib 500 mg/day	22	The ORR was 30.4%. The median OS and PFS were 13.8 and 8.7 months, respectively	(22)
Patients after resection of HCC with PVTT	2020	Apatinib 500 mg/day	30	The median RFS was 7.6 months. The 1-year PFS rate and 1-year OS rate were 36.1% and 93.3%, respectively	(23)
Advanced HCC	2020	Camrelizumab 200 mg every 2 weeks plus apatinib 250 mg/day	190	The ORR was 34.3% and 22.5% in the first- and second-line cohorts, respectively. The median PFS in the cohorts was 5.7 and 5.5 months, respectively. The 12-month survival rate was 74.7% and 68.2%, respectively	(24)
Advanced primary liver cancer after prior systemic treatment(s)	2021	Camrelizumab 200 mg every 2 weeks plus apatinib 125–500 mg/day	28	The ORR was 10.7%. The median PFS and OS were 3.7 and 13.2 months, respectively	(25)
Advanced HCC refractory or intolerant to at least one line of systemic chemotherapy or targeted therapy	2021	Apatinib 750 mg or placebo orally once daily	393	The median OS was significantly improved in the apatinib group compared with that in the placebo group (median 8.7 *vs* 6.8 months)	(26)

HCC, hepatocellular carcinoma; TACE, transcatheter arterial chemoembolization; PFS, progression-free survival; ORR, objective response rate; OS, overall survival; PVTT, portal vein tumor thrombosis; SBRT, stereotactic body radiotherapy.

### Lung Cancer

In addition to the two approved indications above, lung cancer is the indication with the largest number of apatinib-related clinical trials (**Table 3**). In 2018, a phase II clinical trial showed that apatinib had some efficacy with an objective response rate (ORR) of 13.2% and a median PFS of 3.06 months, and manageable toxicity in patients with advanced non-squamous non-small-cell lung cancer (NSCLC) (28). Although this trial revealed only a modest efficacy of apatinib against NSCLC, this drug has been increasingly tested in lung cancer. Results from the next three phase II clinical trials suggested that apatinib exhibited efficacy and an acceptable safety profile in previously heavily treated extensive-stage small-cell lung cancer (SCLC) patients. Further exploration of apatinib in phase III trials is warranted (30, 31, 34). Another phase II clinical trial reported that low-dose apatinib monotherapy might be an option for patients with advanced lung squamous cell carcinoma (33). These clinical trial results indicated that apatinib exerted some clinical activity against all lung cancer subtypes but did not have an advantage over several other lung cancer treatment agents. Owing to the limited efficacy of apatinib as a single agent in lung cancer, an increasing number of clinical trials have begun to investigate apatinib plus alternative agents or therapies, the most common of which is apatinib in combination with chemotherapy. These clinical trials included a phase I trial of apatinib plus docetaxel in advanced lung adenocarcinoma (29); a multicenter, phase II trial of apatinib plus docetaxel in advanced non-squamous NSCLC (35); a phase II trial of apatinib in combination with pemetrexed-platinum chemotherapy in chemo-naive non-squamous NSCLC (36); a randomized, controlled, multicenter clinical trial of apatinib plus docetaxel or pemetrexed in unresectable NSCLC (37); a nonrandomized clinical trial of apatinib plus vinorelbine in wild-type advanced NSCLC (38); and an open-label, multicenter, single-arm phase II trial of apatinib with etoposide capsules in extensive-stage SCLC (41). These studies conclude that apatinib combined with chemotherapy shows promising efficacy and manageable toxicity in enrolled patients. Apatinib plus chemotherapy seemed to be more effective than apatinib alone, and there was no significant increase in toxicity. However, as there are no randomized controlled clinical trials, we cannot identify the difference between the combination regimen and the monotherapy regimen. In addition, phase II and III trials have shown that apatinib plus gefitinib as first-line therapy resulted in superior PFS to placebo plus gefitinib in advanced EGFR-mutant NSCLC. Combination therapy resulted in more adverse events but did not interfere with the quality of life of patients (39, 42). Finally, combined apatinib and camrelizumab showed encouraging antitumor activity and acceptable toxicity in both advanced SCLC (32) and non-squamous NSCLC patients (40). These results suggest that apatinib has great potential in combination with other targeted or immunological agents. In conclusion, apatinib monotherapy has shown activity in all subtypes of lung cancer; however, its efficacy is inferior to that of other established therapies. Currently, apatinib can only be used in combination with other therapies as a treatment option for patients with advanced lung cancer who have failed multiline therapy. In the treatment of advanced lung cancer, the combination of apatinib with other drugs or treatment regimens is the current and next stage of research.

**Table 3:** Clinical trials on apatinib for lung cancer treatment included in this review.

Type of disease	Year of publication	Drugs or therapies	Number of patients	Clinical outcome	References
Pretreated advanced non-squamous NSCLC	2018	Apatinib 750 or 500 mg/day	40	The ORR was 13.2%, the median PFS was 3.06 months, and the median OS was 7.69 months	(28)
Advanced lung adenocarcinoma	2019	Apatinib 250-500 mg/day and docetaxel 60 mg/m^2^ every 3 weeks	12	The median PFS was 2.76 months	(29)
Extensive-stage SCLC	2019	Apatinib 250 mg/day during the chemotherapy interval, and as maintenance therapy after 4–6 cycles	24	The median PFS was 7.8 and 4.9 months in the combination and chemotherapy groups, respectively. The median OS was 12.1 and 8.2 months in the combination and chemotherapy groups, respectively	(30)
Extensive-stage SCLC after two or three previous treatments, including a platinum-based regimen	2019	Apatinib 500 mg/day	40	The ORR was 18.4%, the median PFS was 3.0 months, and the median OS was 5.8 months	(31)
Extensive-stage SCLC	2020	Apatinib 375 mg/day plus camrelizumab 200 mg every 2 weeks	47	The ORR was 34.0%, the median PFS was 3.6 months, and the median OS was 8.4 months	(32)
Advanced lung squamous cell carcinoma	2020	Apatinib 250 mg/day	36	The ORR was 16.7%, the median PFS was 4.9 months, and the median OS was 6.9 months	(33)
Extensive-stage SCLC after failure of two or more lines of chemotherapy	2020	Apatinib 500 mg/day	22	The ORR was 13.6%, the median PFS was 5.4 months, and the median OS was 10.0 months	(34)
Advanced non-squamous NSCLC with wild-type EGFR	2020	Apatinib 500 mg/day plus intravenous docetaxel (60 mg/m^2^ at day 1 every 3 weeks for 4–6 cycles)	29	The ORR was 27.6%, the median PFS was 5.3 months, and the median OS was 9.6 months	(35)
Chemo-naive non-squamous NSCLC	2020	Apatinib 250 mg/day with intravenous pemetrexed (500 mg/m^2^)-platinum (carboplatin AUC = 5 or cisplatin 75 mg/m^2^) chemotherapy every 21 days for six treatment cycles, followed by maintenance with apatinib 250 mg once daily	20	The ORR was 80%, the median PFS was 7.7 months, and the median OS was 20.1 months	(36)
Unresectable locally advanced or advanced NSCLC without driver mutations that had progressed following first-line chemotherapy	2020	Apatinib combined with four cycles of docetaxel or pemetrexed	33	The ORR was 27%, and the median PFS was 5.47 months	(37)
Wild-type advanced NSCLC after second-line treatment failure	2020	Apatinib 500 mg/day and oral vinorelbine 60 mg/m^2^ once weekly	30	The ORR was 36.7%, the median PFS was 4.5 months, and the median OS was 10.0 months	(38)
Advanced non-squamous NSCLC harboring EGFR 19 deletion or 21 L858R point mutation	2020	Cohort A (apatinib 500 mg + gefitinib 250 mg) *vs* Cohort B (apatinib 250 mg + gefitinib 250 mg)	13	Of the 11 patients evaluable for efficacy, Cohort A achieved an ORR of 80.0% and reached a median PFS of 19.2 months, whereas Cohort B attained an ORR and median PFS of 83.3% and 13.4 months, respectively	(39)
Advanced non-squamous NSCLC previously treated with chemotherapy	2020	Apatinib 250–500 mg/day in combination with intravenous camrelizumab 200 mg every 2 weeks	105	In the efficacy-evaluable population (n = 94), the ORR was 30.9%, the median PFS was 89 5.7 months, and the overall survival was 15.5 months	(40)
Extensive-stage SCLC that had progressed following two to three previous therapies	2021	Apatinib (250 mg/day continuously) and etoposide capsules (50 mg/day, on days 1–21, per 28 days)	53	The ORR was 20.8%, the median PFS was 3.0 months, and the median OS was 5.0 months	(41)
Advanced EGFR-mutant NSCLC	2021	A+G group (apatinib 500 mg/day plus gefitinib 250 mg/day) *vs* P+G group (placebo plus gefitinib 250 mg/day)	313	The median PFS was 13.7 *vs* 10.2 months in the A+G group *vs* the P+G group, respectively	(42)

NSCLC, non-small-cell lung cancer; ORR, objective response rate; PFS, progression-free survival; OS, overall survival.

### Sarcoma

Pazopanib was approved by the US Food and Drug Administration in 2014 for increased indications of advanced soft tissue sarcoma (STS) (43). This event inspired clinical researchers in China to apply apatinib, the first domestically produced multitarget TKI similar to pazopanib, in the treatment of sarcoma (**Table 4**). In 2019, the first phase II clinical trial to report results demonstrated the activity (with an ORR of 15.25%) and safety of apatinib in the treatment of advanced STSs (44). Another phase II clinical trial showed that apatinib was a sensitive drug for advanced osteosarcoma, with an ORR of 43.24% after the failure of chemotherapy, with a similar duration of response compared to other TKIs (45). Although the results of these two clinical trials have been somewhat controversial, apatinib is of great interest in the treatment of osteosarcoma and STSs because there are fewer drugs available for advanced sarcomas. In 2020, another phase II trial showed that apatinib showed good efficacy in advanced STS patients, with an ORR of 23.68% (47). This result evidences the efficacy of apatinib in treating STSs. However, there are more than 70 types of STSs, and different subtypes of STSs respond differently to apatinib. Therefore, it is necessary to validate the efficacy of apatinib in different subtypes. Notably, a phase II trial showed that the combination of apatinib and a PD-1 inhibitor did not prolong PFS in comparison to single-agent apatinib in treating advanced osteosarcoma (48), which suggests that the efficacy of apatinib plus PD-1 inhibitors in osteosarcoma is very limited. Nevertheless, although the results of clinical trials of apatinib plus PD-1 inhibitor in STSs are not yet available, based on current evidence, such a combination regimen is desirable (49, 50). In conclusion, apatinib represents a significant development in the treatment of osteosarcoma and STSs, although it is less effective than first-line and second-line chemotherapy. A better therapeutic scenario for apatinib in the treatment of sarcomas remains to be developed.

**Table 4:** Clinical trials on apatinib for sarcoma treatment included in this review.

Type of disease	Year of publication	Drugs or therapies	Number of patients	Clinical outcome	References
Advanced sarcoma after chemotherapy failure	2019	Apatinib 500 mg/day	59	The ORR was 15.25%, and the median PFS was 7.93 months	(44)
Advanced osteosarcoma after failure of standard multimodal therapy	2019	Apatinib 500–750 mg/day	37	The ORR was 43.24%, the median PFS was 4.50 months, and the median OS was 9.87 months	(45)
Advanced chordoma	2020	Apatinib 500 mg/day	30	The ORR was 3.7%, and the median PFS was 18 months	(46)
Advanced STS after the failure of adriamycin-based chemotherapy	2020	Apatinib 500 mg/day	42	The ORR was 23.68%, the median PFS was 7.87 months, and the median OS was 17.55 months	(47)
Advanced osteosarcoma progressing after chemotherapy	2020	Apatinib 500 mg/day plus camrelizumab 200 mg every 2 weeks	43	The ORR was 20.9%, and the 6-month PFS rate was 50.9%	(48)

ORR, objective response rate; PFS, progression-free survival; OS, overall survival; STS, soft tissue sarcoma.

### Breast Cancer

Clinical trials of apatinib in breast cancer began early (**Table 5**). In 2014, multicenter phase II trials showed that apatinib exhibited objective efficacy and manageable toxicity in heavily pretreated metastatic non-triple-negative breast cancer (NTNBC) (with an ORR of 16.7%) (51) and in TNBC (with an ORR of 10.7%) (52). Although these two clinical trials initially showed that the activity of apatinib in breast cancer was similar to that of other multitarget TKIs of the same class, the efficacy of apatinib was considered limited in breast cancer in comparison to a large number of established therapies (51). Therefore, the activity of apatinib monotherapy against breast cancer has not been further tested. Hopes are pinned on the combination of apatinib and other drugs. In 2020, a phase II trial showed that apatinib combined with etoposide capsules is effective and tolerable in heavily pretreated, metastatic HER2-negative breast cancer patients (53). Another phase II trial showed that the ORR of an apatinib plus PD-1 inhibitor regimen was markedly higher than the previously reported ORR of anti-PD-1/PD-L1 antibody or apatinib monotherapy in advanced TNBC (54). Two recent clinical trials have shown that apatinib is active and safe in combination with endocrine therapy or oral vinorelbine for the treatment of HER2-negative breast cancer (55, 56). These four clinical trials with small sample sizes preliminarily proved that apatinib combined with other drugs has promising activity in the treatment of breast cancer. These combination regimens should be validated in large, controlled, prospective clinical trials. In conclusion, the efficacy of apatinib monotherapy in breast cancer is assumed to be limited, and the efficacy of apatinib in combination with other drugs or therapies needs to be further tested.

**Table 5:** Clinical trials on apatinib for breast cancer treatment included in this review.

Type of disease	Year of publication	Drugs or therapies	Number of patients	Clinical outcome	References
Heavily pretreated advanced non-TNBC	2014	Apatinib 500 mg/day	38	The ORR was 16.7%, the median PFS was 4.0 months, and the median OS was 10.3 months	(51)
Heavily pretreated metastatic TNBC	2014	Apatinib 500 mg/day	56	The ORR was 10.7%, the median PFS was 3.3 months, and the median OS was 10.6 months	(52)
Heavily pretreated metastatic breast cancer	2020	Apatinib 450–500 mg/day, and etoposide capsules 50 mg/m^2^ on days 1 to 10 for 21 days	31	The ORR was 35.5%, the median PFS was 6.9 months, and the median OS was 20.4 months	(53)
Advanced TNBC after failure of less than three lines of systemic therapy	2020	Apatinib 250 mg/day plus camrelizumab 200 mg every 2 weeks	30	The ORR was 43.3%, and the median PFS was 3.7 months	(54)
HER2-negative breast cancer involving chest wall metastasis	2021	Apatinib 500 mg/day	26	The ORR was 42.3%, the median PFS was 4.9 months, and the median OS was 18 months	(55)
Heavily pretreated HER2-negative metastatic breast cancer	2021	Apatinib 500 mg/425 mg daily plus oral vinorelbine 60 mg/m^2^ on days 1, 8, and 15 of every cycle	40	The ORR was 17.1%, the median PFS was 5.2 months, and the median OS was 17.4 months	(56)

TNBC, triple-negative breast cancer; ORR, objective response rate; PFS, progression-free survival; OS, overall survival.

### Esophageal Cancer

In 2020, a phase II trial showed that apatinib was effective as second- or further-line treatment for advanced esophageal cancer, with an ORR of 7.7% (2/26) (57); another clinical trial involving 40 patients reported similar results (58). The results of these two clinical trials suggested that apatinib monotherapy is not ideal for the treatment of advanced esophageal cancer (**Table 6**). In another phase II clinical trial, apatinib plus PD-1 inhibitor combined with liposomal paclitaxel and nedaplatin as a first-line treatment showed antitumor activity (with an ORR of 80.0%) and manageable safety in patients with advanced esophageal cancer (59). However, it is not possible to make definite interpretations regarding the contribution of apatinib in this complex combination of treatments. In conclusion, based on current evidence, apatinib has moderate efficacy against advanced esophageal squamous cell carcinoma, and clinical trials with large sample sizes are needed to identify the optimal setting for the use of apatinib in esophageal cancer.

**Table 6:** Clinical trials on apatinib for the treatment of malignant tumors included in this review.

Type of disease	Year of publication	Drugs or therapies	Number of patients	Clinical outcome	References
Advanced ESCC or adenocarcinoma of the esophagus or esophagogastric junction	2020	Apatinib 500 mg/day	26	The ORR was 7.7%, the median PFS was 4.63 months, and the median OS was 6.57 months	(57)
Unresectable locally advanced or recurrent/metastatic ESCC	2020	Camrelizumab 200 mg, liposomal paclitaxel 150 mg/m^2^, and nedaplatin 50 mg/m^2^ on day 1, and apatinib 250 mg on days 1–14. The treatments were repeated every 14 days for up to nine cycles, followed by maintenance therapy with camrelizumab and apatinib	30	The ORR was 80.0%, the median PFS was 6.85 months, and the median OS was 19.43 months	(59)
Chemotherapy- refractory advanced or metastatic ESCC	2021	Apatinib 500 mg/day	40	The ORR was 7.5%, the median PFS was 3.8 months, and the median OS was 5.8 months	(58)
Adenocarcinoma of the colon or rectum after at least two prior regimens of standard therapies including fluoropyrimidine, oxaliplatin, and irinotecan	2019	Apatinib 500 mg/day	26	The median PFS was 3.9 months, and the median OS was 7.9 months	(60)
Microsatellite-stable metastatic colorectal cancer	2020	Camrelizumab 200 mg every 2 weeks and apatinib 250–375 mg/day	10	The ORR was 0%, the median PFS was 1.83 months, and the median OS was 7.80 months	(61)
Metastatic colorectal cancer after failure of two or more lines of standard fluorouracil-based chemotherapy	2020	Apatinib 500 mg/day	48	The ORR was 8.3%, the median PFS was 4.8 months, and the median OS was 9.1 months	(62)
Platinum-resistant or platinum-refractory ovarian cancer	2018	Apatinib 500 mg/day on a continuous basis, and oral etoposide at a dose of 50 mg once daily on days 1–14 of a 21-day cycle. Oral etoposide was administered for a maximum of six cycles	35	The ORR was 54%	(63)
Advanced cervical cancer that had progressed after at least one line of systemic therapy	2020	Camrelizumab 200 mg every 2 weeks and apatinib 250 mg/day	45	The ORR was 55.6%, and the median PFS was 8.8 months	(64)
Advanced cholangiocarcinoma after refractory chemotherapy	2021	Apatinib 500 mg/day	26	The ORR was 11.5%, the median PFS was 2.0 months, and the median OS was 9.0 months	(65)
Relapsed or refractory diffuse large B-cell lymphoma	2020	Apatinib 500 mg/day	32	The ORR was 43.8%, the median PFS was 6.9 months, and the median OS was 7.9 months	(66)
Metastatic or locoregionally recurrent nasopharyngeal carcinoma after refractory of chemotherapy	2021	Apatinib 500 mg/day	33	The ORR was 36.4%, the median PFS was 5 months, and the median OS was 16 months	(67)
Progressive radioiodine-refractory differentiated thyroid cancer	2021	Apatinib 500 mg/day	20	The ORR was 80%, the median PFS was 18.4 months, and the median OS was 51.6 months	(68)

ESCC, esophageal squamous cell carcinoma; ORR, objective response rate; PFS, progression-free survival; OS, overall survival.

### Colorectal Cancer

In 2019, a phase II trial showed that apatinib monotherapy exerted promising efficacy in patients with refractory colorectal cancer, with a median PFS of 3.9 months (**Table 6**) (60). In another trial, apatinib monotherapy exhibited encouraging efficacy (with an ORR of 8.3% and a median PFS of 4.8 months) with manageable toxicities in chemotherapy-refractory metastatic colorectal cancer (62). These results indicate that apatinib has a certain efficacy against colorectal cancer, which is worthy of further verification in clinical trials with larger sample sizes. However, results from another phase II clinical trial, already published in 2020, showed that combination with a PD-1 inhibitor failed to improve the efficacy of apatinib in treating metastatic colorectal cancer (61). The researchers concluded that this situation was caused by insufficient effective doses due to the high toxicity of the combination therapy. In conclusion, apatinib may have moderate activity against colorectal cancer. Clinical trials with large sample sizes are needed to determine whether apatinib is more efficacious as a single agent or in combination with other agents for the treatment of advanced colorectal cancer.

### Other Cancers

In 2018, a phase II trial showed that the combination of apatinib with oral etoposide showed promising efficacy (with an ORR of 54.3%) and manageable toxicities in patients with platinum-resistant or platinum-refractory ovarian cancer (**Table 6**) (63). However, the study had a small sample size, and the significance of apatinib in this combination regimen is unclear (69). The activity of apatinib in ovarian cancer needs to be further validated in clinical trials with large sample sizes. As in ovarian cancer, only one phase II clinical trial has reported the activity of apatinib in advanced cervical cancer. This clinical trial showed that apatinib plus PD-1 inhibitor showed promising antitumor activity (with an ORR of 55.6%) and manageable toxicities in patients with advanced cervical cancer (64). Larger randomized controlled trials are warranted to validate these findings.

In addition, several recent phase II clinical trials have confirmed that apatinib monotherapy has certain efficacy and good safety in the treatment of advanced cholangiocarcinoma (ORR, 11.5%) (65), relapsed or refractory diffuse large B-cell lymphoma (ORR, 43.8%) (66), metastatic or locoregionally recurrent nasopharyngeal carcinoma (ORR, 36.4%) (67), and radioiodine-refractory differentiated thyroid cancer (ORR, 80%) (68). These clinical trials have only initially shown the efficacy and safety of apatinib in these malignancies (**Table 6**). Therefore, we cannot conclude the true efficacy of apatinib in these malignancies owing to the small sample sizes of these single-arm clinical trials. Extensive clinical trials are needed to confirm the efficacy and optimal use of apatinib in these malignancies.

## Biomarkers of Response to Apatinib in Different Malignancies

In addition to efficacy and safety in various malignancies, response biomarkers are an important topic in clinical research of apatinib. We searched all literature related to potential biomarkers of the antitumor efficacy of apatinib in the treatment of malignancies and screened out all possible efficacy biomarkers currently reported. The results are presented in **Tables 7** and **8**.

**Table 7:** Response biomarkers of apatinib associated with improved outcomes in the treatment of malignancies.

Tumor species studied	Biomarker Name	Drugs or therapies	Conclusion	Reference
Breast cancer	P-VEGFR2, hypertension	Apatinib	P-VEGFR2 and hypertension were independent predictive factors for both PFS and clinical benefit rate.	(70)
Breast cancer	No gene variant detected and lower variant allele frequencies in ctDNA at baseline	Apatinib and vinorelbine	Patients with no gene variant detected and lower variant allele frequencies in ctDNA at baseline showed longer PFS.	(56)
Breast cancer	Hypertension and proteinuria	Apatinib and oral etoposide	The median PFS of patients who had hypertension and proteinuria was longer than that of those without hypertension and proteinuria.	(53)
Breast cancer	High percentage of baseline TILs	Apatinib and camrelizumab	High percentage of baseline TILs (>10%) was associated with higher ORR and favorable PFS.	(54)
Breast cancer	Higher baseline TILs or a greater increase of tumor-infiltrating CD8+ T cells during therapy, lower baseline plasma HGF/IL-8, a decrease of plasma IL-8, an increase of plasma TIM-3/CD152 during therapy, higher baseline CD4+ T cells or B cells proportion in blood	Apatinib and camrelizumab	Higher baseline TILs or a greater increase of tumor-infiltrating CD8+ T cells during therapy, lower baseline plasma HGF/IL-8, a decrease of plasma IL-8, an increase of plasma TIM-3/CD152 during therapy, higher baseline CD4+ T cells or B cells proportion in blood are potential biomarkers associated with better outcomes.	(71)
Cervical Cancer	Genetic alterations in PIK3CA, PTEN, ERBB3, and PI3K/AKT pathway, as well as TMB.	Apatinib and Camrelizumab	Genetic alterations in PIK3CA, PTEN, PI3K/AKT pathway, and TMB were associated with improved outcomes.	(72)
Colorectal cancer	Neutrophil/lymphocyte ratio, carbohydrate antigen 19–9, and HFS	Apatinib	Low baseline neutrophil/lymphocyte ratio, early carbohydrate antigen 19–9 decrease, and HFS were associated with improved PFS.	(62)
Gastric cancer	Hypertension, proteinuria, HFS	Apatinib	Presence of hypertension, proteinuria, or HFS during the first cycle of apatinib treatment was a viable biomarker of antitumor efficacy in metastatic gastric cancer patients.	(73)
Gastric cancer	Hypertension, proteinuria and/or HFS	Apatinib/paclitaxel	The occurrence of hypertension, proteinuria and/or HFS were independent factors associated with better survival outcomes	(7)
Gastric cancer	Leukopenia, and HFS	Apatinib and/or taxel/docetaxel	The potential biomarkers associated with longer PFS were occurrence of leukopenia and HFS.	(8)
Gastric cancer and lung cancer	Lung cavitation	Apatinib	Lung cavitation development was beneficial in patients receiving apatinib therapy regardless of whether they had primary or metastatic lung cancer.	(74)
HCC	PLR	Apatinib plus transarterial chemoembolization	The median PFS and OS in the PLR ≤150 group were longer than those in the PLR >150 group.	(75)
HCC	Hypertension	Apatinib	Apatinib-related hypertension can potentially predict prolonged survival.	(76)
HCC	The clinical-radiomics nomograms	Apatinib plus transarterial chemoembolization	The clinical-radiomics nomograms, a noninvasive pretreatment prediction tool that incorporate radiomics signature and AFP, demonstrated good prediction accuracy for OS and PFS in these patients.	(77)
HCC	LMR, PNI	Apatinib and Camrelizumab	The remission rate in patients with high LMR was higher than that in patients with low LMR, and the remission rate in patients with high PNI was higher than that in patients with low PNI.	(78)
NSCLC	Hypertension	Apatinib	Hypertension was independently associated with improved PFS and OS on both univariate and multivariate analyses.	(79)
NSCLC	STK11/KEAP1 mutation	Apatinib and Camrelizumab	Patients with STK11/KEAP1 mutation might derive more benefits from this combination.	(40)
Ovarian cancer	Adipose tissue area	Apatinib and oral etoposide	High Adipose tissue area were significantly associated with better outcomes	(80)
Osteosarcoma	Anorexia, hypertension, pneumothorax, and hypothyroidism	Apatinib	Anorexia, hypertension, pneumothorax, and hypothyroidism might be markers for a favorable clinical outcome following apatinib- treated refractory osteosarcoma.	(81)
Osteosarcoma	Pneumothorax and cavitation in lung metastases	Apatinib	Pneumothorax and cavitation in lung metastases may be effective prognostic markers.	(82)
Sarcoma	Hypertension, proteinuria, HFS	Apatinib	The development of hypertension, HFS, or proteinuria may indicate a favorable prognosis.	(44)
Sarcoma	Hypertension, proteinuria, HFS	Apatinib	The subjects who experienced hypertension, HFS, or proteinuria had significantly longer OS than those without these AEs	(47)
Thyroid cancer	BRAFV600E mutation	Apatinib	Patients with BRAFV600E mutation had a longer median PFS compared with patients with BRAF wild-type.	(68)

AE, adverse event; P-VEGFR2, Phosphorylated vascular endothelial growth factor receptor 2; HFS, hand and foot syndrome; PFS, progression-free survival; TILs, tumor-infiltrating lymphocytes; ORR, objective response rate; OS, overall survival; NSCLC, non-small-cell lung cancer; HCC, hepatocellular carcinoma; PLR, platelet-to-lymphocyte ratio; LMR, lymphocyte-to-monocyte ratio; PNI, prognostic nutritional index.

**Table 8:** Response biomarkers of apatinib associated with poor outcomes in the treatment of malignancies.

Tumor species studied	Biomarker Name	Drugs or therapies	Conclusion	Reference
Breast cancer	Gene variants in ctDNA	Apatinib in combination with oral vinorelbine	Patients with no gene variant detected and lower variant allele frequencies in ctDNA at baseline showed longer PFS.	(56)
Cervical Cancer	ERBB3 mutations	Apatinib and Camrelizumab	ERBB3 mutations correlated with poor survival.	(72)
Gastric cancer	CEA elevation	Apatinib	CEA elevation was considered to be a potential independent predictive factor associated with shorter PFS and OS.	(13)
Nasopharyngeal carcinoma	EBV DNA titer	Apatinib	High EBV DNA titer were significant prognostic factors associated with shorter PFS.	(83)
Non-small- cell lung cancer	cfDNA concentration, MIKI67 mutations and HPD-related mutations	Apatinib and Camrelizumab	High cfDNA concentration, MIKI67 mutations and HPD-related mutations were independent risk factors and worse PFS predictors for apatinib and camrelizumab combined therapy.	(84)
Non-squamous non-small cell lung cancer	Presence of CNS metastasis at baseline	Apatinib in combination with pemetrexed-platinum chemotherapy	The mPFS in the responders without CNS metastasis at baseline was significantly longer than that in those with presence of CNS metastasis at baseline.	(36)

PFS, progression-free survival; OS, overall survival; EBV DNA, Epstein-Barr virus DNA; CNS, central nervous system.

As shown in **Tables 7** and **8**, several potential biomarkers have been reported to predict the response of patients to apatinib. According to the predicted outcomes, these biomarkers can be classified as those associated with improved outcomes (**Table 7**) or those associated with poor outcomes (**Table 8**). Based on their properties, these biomarkers can be divided into three categories: adverse event markers, routine test markers, and genetic test markers. Adverse event markers include hypertension, proteinuria, hand and foot syndrome (HFS), lung cavitation, anorexia, pneumothorax, and hypothyroidism. Routine test markers include phosphorylated VEGFR2, higher baseline TILs or a greater increase in tumor-infiltrating CD8+ T cells during therapy, lower baseline plasma HGF/IL-8, a decrease in plasma IL-8, an increase in plasma TIM-3/CD152 during therapy, higher baseline CD4+ T-cell or B-cell proportion in blood, leukopenia, platelet-to-lymphocyte ratio, lymphocyte-to-monocyte ratio, prognostic nutritional index, clinical-radiomics nomograms, adipose tissue area, neutrophil-to-lymphocyte ratio, and carbohydrate antigen 19–9. Genetic test markers include cfDNA concentration, MIKI67 mutations, HPD-related mutations, STK11/KEAP1, PIK3CA, PTEN, ERBB3, PI3K/AKT, and TMB mutations.

Nevertheless, owing to high technical requirements and cost, it is difficult to screen for genetic test biomarkers on a large scale in clinical practice. In contrast, adverse event and routine test biomarkers are highly effective and can be easily implemented in various diagnostic and treatment institutions on a large scale. However, although many potential prognostic markers of apatinib have been reported, none of them have been widely used in clinical practice. The main reasons include the following: First, the level of the currently available evidence is not high; current studies on the potential biomarkers of apatinib are either retrospective studies or adjunct to efficacy and safety trials, and only a few prospectively registered trials have focused on prognostic biomarkers. Second, prognostic biomarker studies are still in the exploratory stage. Significant differences in efficiency were observed between different types of markers in various malignancies. Third, the prediction efficiency of individual markers is not high, leading to a lack of value for these markers in the clinical setting. The combined prediction of multiple markers may be needed to improve their predictive efficiency and clinical application value. What is exciting is that some researchers have tried to model this type of joint prediction (77).

In conclusion, there are still many problems to be solved in the study of apatinib-related prognostic biomarkers, and prospectively registered trials with large sample sizes aimed to identify specific markers should be conducted.

## Discussion

To the best of our knowledge, this review is the first to summarize the efficacy and prognostic biomarkers of apatinib in various malignancies over the last 3 years. In this review, we first summarized the efficacy of apatinib against various malignancies in clinical trials where results have been reported. Apatinib is currently approved in China for the treatment of advanced GC and HCC. In addition to these two malignancies, apatinib has been studied in detail against lung cancer, breast cancer, and sarcoma. Apatinib has a great application value for the treatment of these tumor species. Moreover, the efficacy of apatinib against esophageal cancer, colorectal cancer, ovarian cancer, cervical cancer, cholangiocarcinoma, diffuse large B-cell lymphoma, nasopharyngeal carcinoma, and differentiated thyroid cancer has been proven in prospectively registered trials. Additionally, we reviewed the current literature on biomarkers of apatinib response. According to their properties, these biomarkers can be divided into three categories: adverse event, routine test, and genetic test markers. The findings of this review have a good reference value for the application of apatinib in clinical studies and the design of clinical trials.

This review identified some problems and phenomena in apatinib treatment. The first is the narrow range of apatinib monotherapy scenarios. As a monotherapy regimen, apatinib may be difficult to be approved for additional cancer indications. Long-term, off-label use of apatinib may be required for the treatment of malignancies other than GC and HCC. Second, clinical trials of apatinib in combination with other drugs or therapies are still in the early stages of development. It seems that apatinib can be used in combination with other anticancer therapies. Currently, the most studied is the combination of apatinib and camrelizumab (a PD-1 inhibitor from the same manufacturer). Studies on apatinib in combination with common chemotherapeutic agents are also increasing. We predict that the next approved clinical indication for apatinib is likely to be in the form of combination therapy with other drugs or therapies. Third, the optimal scenario for apatinib in most malignancies is unclear. Although apatinib monotherapy is approved for advanced GC and advanced HCC, combination regimens based on apatinib may be more effective. Current evidence suggests that apatinib monotherapy in many malignancies is not a substitute for existing mature regimens, but the optimal use of apatinib in a particular cancer (e.g., as a monotherapy or in combination? Combination with which agent or therapy? As a neoadjuvant or advanced multiline therapy?) is unclear. Finally, the current research on response biomarkers of apatinib is limited. On the one hand, the current level of evidence obtained by apatinib prognostic marker studies is not high. On the other hand, even though many therapeutic markers have been reported and each therapeutic marker has a different predictive value in different tumor species, the overall predictive efficiency is not high.

To solve these problems, the next clinical study of apatinib can follow three major directions. The first is to further expand the use of apatinib in different malignancies. There are hundreds of types of malignant tumors (85), and as a late-marketed multitarget TKI, apatinib has fewer clinical trials compared with other multitarget TKIs in the same class, such as pazopanib and sorafenib (86, 87). Prospective, registered clinical trials of apatinib should be conducted for various malignancies that respond to multitarget TKIs. The second is to continue to expand and determine the best application scenarios of apatinib for specific tumor species. Third, through a systematic study of the available evidence on response biomarkers of apatinib, we established that there are some effective response biomarkers for this drug. However, to improve the level of evidence and our understanding of these biomarkers, prospective registration trials with large sample sizes focusing on specific biomarkers should be conducted.

In conclusion, this review summarized the current knowledge on the efficacy and response biomarkers of apatinib for malignancy treatment, highlighted the current problems to be solved in the clinical application of apatinib, and proposed promising directions for future clinical research. Although much work remains to be done, apatinib will eventually advance from a new targeted drug for many malignancies to a cornerstone drug, similar to cyclophosphamide and doxorubicin among common chemotherapy drugs.

## Author Contributions

All authors listed have made a substantial, direct, and intellectual contribution to the work, and approved it for publication.

## Conflict of Interest

The authors declare that the research was conducted in the absence of any commercial or financial relationships that could be construed as a potential conflict of interest.

## Publisher’s Note

All claims expressed in this article are solely those of the authors and do not necessarily represent those of their affiliated organizations, or those of the publisher, the editors and the reviewers. Any product that may be evaluated in this article, or claim that may be made by its manufacturer, is not guaranteed or endorsed by the publisher.
